# Correlation between uric acid levels and bone mineral density in patients with type 2 diabetes mellitus: a systematic review and meta-analysis

**DOI:** 10.3389/fendo.2025.1415550

**Published:** 2025-02-07

**Authors:** Hang Zhao, Cuijuan Qi, Yunjia Zhang, Luping Ren, Shuchun Chen

**Affiliations:** Department of Endocrinology, Hebei General Hospital, Shijiazhuang, Hebei, China

**Keywords:** type 2 diabetes, bone mineral density, osteoporosis, uric acid, bone metabolism

## Abstract

**Purpose:**

To explore the controversial relationship between uric acid (UA) levels and bone mineral density (BMD) in patients with type 2 diabetes mellitus (T2DM).

**Patients and methods:**

The PubMed, Embase, and Cochrane Library databases were searched using keywords and related words. Study quality was evaluated using the Newcastle-Ottawa Scale. Studies retrieved in the literature search were systematically screened to extract information and data based on predefined inclusion and exclusion criteria. RevMan version 5.3 and Stata Release 13.0 were used for statistical analysis. Results are expressed as mean difference (MD) and corresponding 95% confidence interval (CI). Heterogeneity was evaluated using the I^2^ and Q tests.

**Results:**

This meta-analysis included 10 studies comprising 5,717 patients with T2DM. Study quality ranged from moderate to high. Results of comparative analyses were as follows: normal BMD versus (vs.) osteoporosis (OP) in females, MD −13.83 μmol/L (95% CI −41.69 to 14.03); I^2^ = 7%; P=0.30); normal BMD vs. osteopenia in females, MD −12.41 μmol/L (95% CI −37.81 to 12.99; I^2^ = 0%; P=0.92); normal BMD vs. abnormal BMD (osteopenia/OP), MD −23.82 μmol/L (95% CI −33.50 to −14.13; I^2^ = 0%; P=0.44); and osteopenia vs. OP, MD −22.35 μmol/L (95% CI −29.55 to −15.15; I^2^ = 5%; P=0.39). No publication bias was observed.

**Conclusion:**

Compared with normal BMD, abnormal BMD (osteopenia/OP) was associated with lower UA levels. Compared with osteopenia, OP also showed lower UA.

Systematic review registration:

## 1 Introduction

Bone is a dynamic organization of tissue that constantly undergoes remodeling, balancing the processes of bone formation and absorption, which are regulated by osteoblasts and osteoclasts (1). Osteoporosis (OP) is a chronic bone metabolism disease characterized by a decrease in bone mass, destruction of bone microstructure, and increased risk for fractures due to an imbalance between bone formation and destruction. It is estimated that 200 million individuals are affected by OP worldwide and the incidence of OP is higher among females than in males (2). More than 30% of fractures in the elderly population are associated with OP (3).

OP, as a disease of bone metabolic imbalance, is typically manifested as lower back pain, spinal deformity and fractures. Of, the pain during fractures is more severe. It affects quality of life and often requires bed rest, and even death in rare cases. One pathogenic process of OP is oxidative stress, which can affect the activity of bone cells, metabolic pathways, and the expression of bone metabolism-related factors (4). Serum uric acid (UA) is a powerful scavenger of oxygen and hydroxyl radicals, and is the main antioxidant in the plasma, which can protect cells from oxidative damage (5).

Previous studies have explored the relationship between UA levels and bone mineral density (BMD), although the results have varied. Subjects in the present study included patients diagnosed with type 2 diabetes mellitus (T2DM), which also has an impact on bone metabolism. OP, also known as diabetic OP, is a complication associated with diabetes mellitus (DM). Recent studies have explored the relationship between UA and bone metabolism in this population; however, the results have been inconsistent. As such, this study aimed to explore the relationship among UA levels, BMD, and OP in patients with T2DM.

## 2 Patients and methods

### 2.1 Search strategy

A literature search of the PubMed, Embase, and Cochrane Library databases for relevant studies, published from inception to November 10, 2023, was performed using the keywords, free words, and related words “Osteoporosis”, “Bone Diseases, Metabolic”, “Uric Acid” and “Diabetes Mellitus, Type 2” (**Supplementary Material**).

### 2.2 Study selection

**Figure 1:**
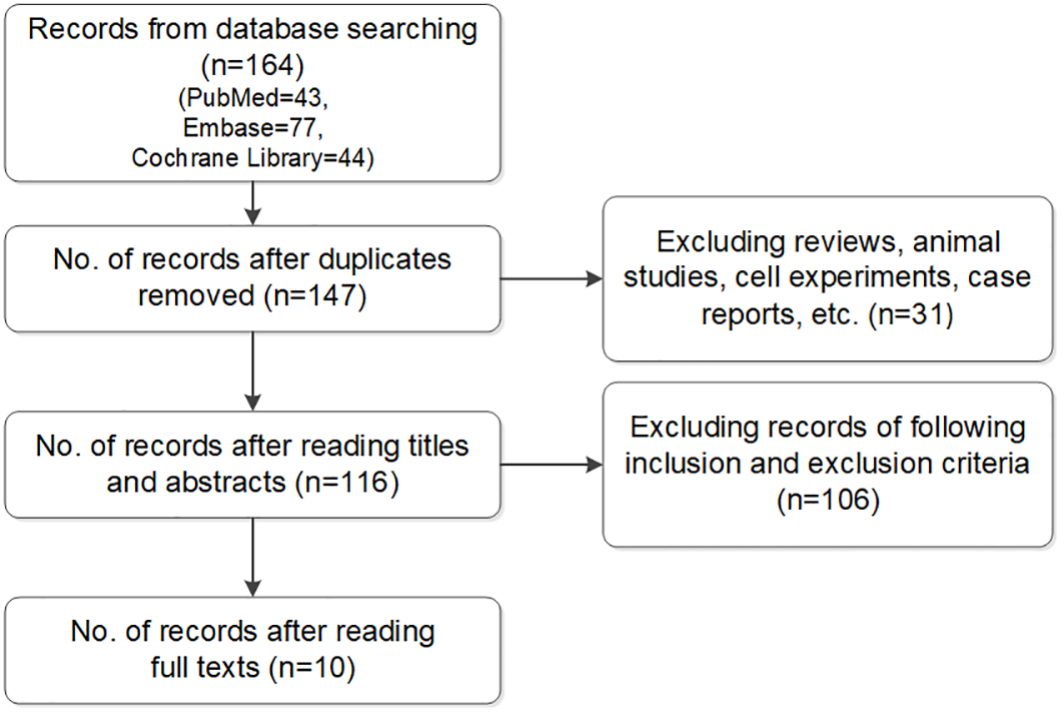
Flow chart of included studies.

Two researchers conducted this process. If there were objections, the studies were discussed with a third one to decide whether to include them. Articles retrieved in the literature search were imported into dedicated software (Endnote, Clarivate, London, United Kingdom) and duplicate studies were identified and excluded. Reviews, meta-analyses, comments, and case reports were excluded after a title screen, while unrelated studies were removed based on title and abstract. The full texts of the remaining studies were reviewed to evaluate their eligibility for inclusion (**Figure 1**).

The inclusion criteria were as follows: observational studies; diagnosis of T2DM; UA data included in the text; and involvement in BMD, osteopenia, and OP. Studies addressing other types of diabetes, combined acute diseases for 3 months, such as cerebral infarction, myocardial infarction, and acute renal injury, chronic kidney disease stage 4 or 5, sarcopenia, severe gouty arthritis, and osteoarthritis, and case reports, reviews, and comments were excluded.

Studies were selected in accordance with the inclusion and exclusion criteria. Disagreements or uncertainties were resolved by consensus discussion between ≥ 2 of the authors.

### 2.3 Data extraction

The following information was extracted from the full-text articles: author(s) name; year of publication; country of origin; age; sex; sample size; and BMD, osteopenia, OP, and UA levels.

### 2.4 Evaluation of study quality

The quality of the included studies was evaluated using the Newcastle-Ottawa Scale (NOS), as follows: poor (1–3 points); moderate (4–6 points); and high (7–9 points). The specific scoring criteria were as follows: selection (adequate case definition, case representativeness, selection of controls, and definition of controls); comparability (comparability of cases and controls based on the design or analysis); and exposure (ascertainment of exposure, identical  method of ascertainment for cases and controls, and non-response rate).

### 2.5 Statistical analysis

Statistical analyses were performed using RevMan version 5.3 and Stata Release 13.0 (StataCorp LLC, College Station, TX, USA). The Mantel–Haenszel statistical model was used, and statistical data are expressed as mean difference (MD) and corresponding 95% confidence interval (CI). I^2^ and Q statistical tests were used to evaluate study heterogeneity. There was no heterogeneity if I^2^ < 50% or P > 0.1 in the Q test. The source of heterogeneity was analyzed using sensitivity analysis, subgroup analysis, or conversion from a fixed-effects model to a random-effects model. Egger’s test was used to evaluate publication bias, with P > 0.05 indicating no publication bias.

## 3 Results

### 3.1 Literature search and screening

The literature search of the 3 databases retrieved 164 articles: PubMed, n=43; Embase, n=77; and Cochrane Library, n=44. Seventeen duplicate articles and 31 case reports, reviews, and comments were excluded based on screening the titles and abstracts. The full texts were read and screened in accordance with the inclusion and exclusion criteria. Ultimately, 10 studies were included in the meta-analysis (6–15).

### 3.2 Characteristics of the included studies

The publication year of the included studies ranged from 2017 to 2023, among which 5,717 patients with T2DM were included. Detailed information is summarized in **Table 1**.

**Table 1:** Characteristics of studies of included.

First author	Publication year	Country	Age	Sex	Sample size	Comparison
Dai (6)	2022	China	60.8 ± 7.28	M	418	(1) OP vs. normal BMD: lower UA in OP group (2) OP vs. osteopenia: lower UA in OP group (3) Osteopenia vs. normal BMD: lower UA in OP group
Lu (7)	2023	China	59.2 ± 9.7	M, F	251	Abnormal BMD vs. normal BMD: no difference
Pan (8)	2021	China	70.31 ± 9.49	F	601	Non-OP vs. OP: no difference
Wu (9)	2021	China	57.3 ± 12	M	631	Abnormal BMD vs. normal BMD: lower UA in abnormal group
Xu (10)	2023	China	56.8 ± 13.9	M, F	485	Abnormal BMD vs. normal BMD: lower UA in abnormal group
Yan (11)	2018	China	63.87 ± 9.33	M, F	1,562	(1) Normal BMD vs. OP: lower UA in OP group (2) OP vs. osteopenia: lower UA in OP group (3) Osteopenia vs. normal BMD: lower UA in osteopenia group
Zhao (12)	2023	China	63.51 ± 7.79	M, F	1,158	(1) Normal BMD vs. OP: no difference (2) OP vs. Osteopenia: lower UA in OP group in male patients, no difference in female patients (3) Osteopenia vs. normal BMD: no difference
Zhao (13)	2020	China	63.65 ± 7.90	F	262	(1) Normal BMD vs. OP: no difference (2) OP vs. osteopenia: no difference (3) Osteopenia vs. normal BMD: no difference
Zheng (14)	2022	China	57.8 ± 11.7	M, F	250	Non-OP vs. OP: lower UA in OP group
Zhou (15)	2017	China	62 ± 8	F	99	(1) Normal BMD vs. OP: no difference (2) Osteopenia vs. normal BMD: no difference

BMD, bone mineral density; F, female; M, male OP, osteoporosis.

### 3.3 Quality assessment

The distribution of NOS scores among the 10 included studies was as follows: 5 points (n=7); 6 points (n=1); and 7 points (n=2). Overall, the quality of the included studies was acceptable.

### 3.4 Statistical results

The aim of the study was to explore the relationship of UA and bone metabolism status (osteopenia and OP). The main finding results were: (1) In female patients, no difference was existed between the normal BMD group and OP group or osteopenia group regarding UA. (2) UA level was lower in the abnormal BMD group than in the normal BMD group. (3) UA levels were lower in the OP group than in the osteopenia group.

#### 3.4.1 Comparison of UA level in the normal BMD group and OP group

**Figure 2:**
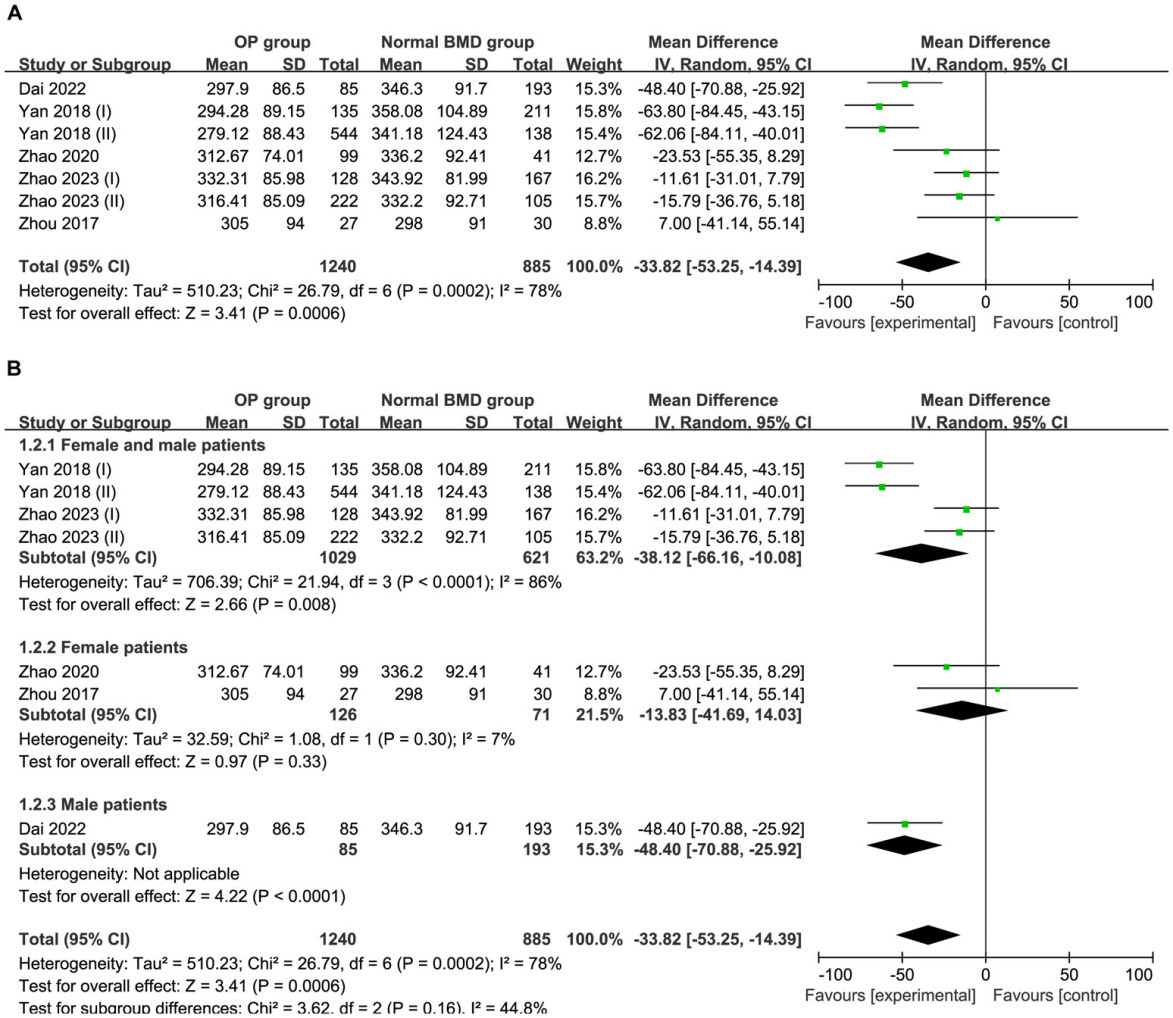
**(A)** Forest of plot comparison of UA level in the normal BMD group and OP group; **(B)** Subgroup analysis based on sex of UA in the normal BMD group and OP group.

Five studies comprising 2,125 patients were included in this meta-analysis. The total effect was MD−33.82 μmol/L (95% CI −53.25 to −14.39; I^2^ = 78%; P=0.0002) using a random effect model, which indicated heterogeneity (**Figure 2A**). A subgroup analysis was performed according to sex. The female and male subgroup had a MD of −38.12 μmol/L (95% CI −66.16 to −10.08; I^2^ = 86%; P<0.00001), the female subgroup had a MD of −13.83 μmol/L (95% CI −41.69 to 14.03; I^2^ = 7%; and P=0.30), indicating no difference between the normal BMD group and OP group regarding UA in females (**Figure 2B**). Egger’s test indicated no publication bias (P=0.231) (**Supplementary Figure S1**).

#### 3.4.2 Comparison of UA level in normal BMD group and osteopenia group

**Figure 3:**
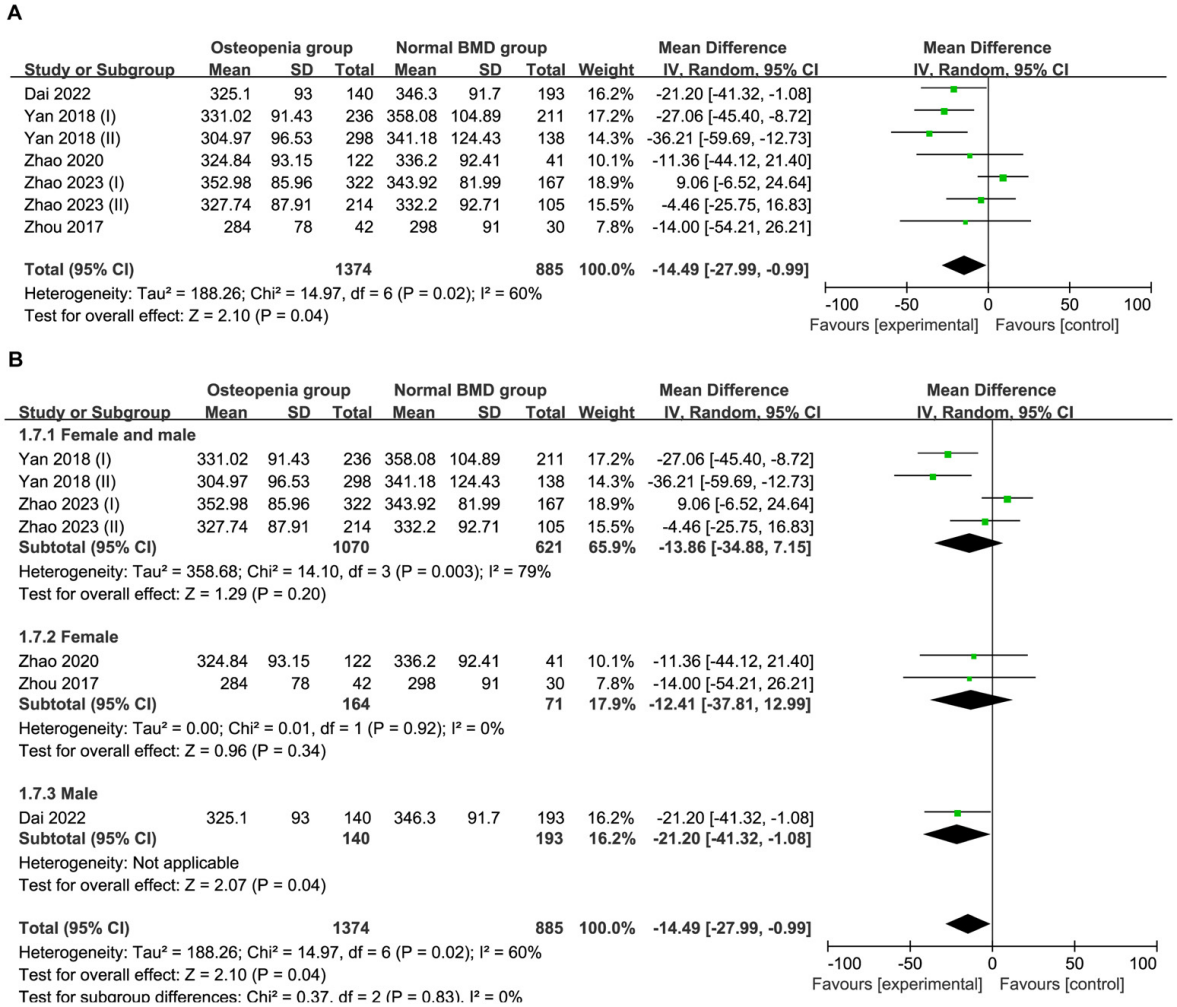
**(A)** Forest of plot comparison of UA level in normal BMD group and osteopenia group; **(B)** Subgroup analysis based on sex of UA in the normal BMD group and osteopenia group.

Six studies comprising 2,259 patients were included in this meta-analysis. The total effect was MD−14.49 μmol/L (95% CI −27.99 to −0.99; I^2^ = 60%; P=0.02) using a random effect model, which indicated heterogeneity (**Figure 3A**). A subgroup analysis was performed according to sex. The female and male subgroup had an MD of −13.86 μmol/L (95% CI −34.88 to 7.15; I^2^ = 79%; and P=0.003), the female subgroup had a MD −12.41 μmol/L (95% CI −37.81 to 12.99; I^2^ = 0%; P=0.92), indicating no difference between the normal BMD group and the osteopenia group regarding UA levels in females (**Figure 3B**). Egger’s test indicated no publication bias (P=0.994) (**Supplementary Figure S2**).

#### 3.4.3 Comparison of UA level in normal BMD group and abnormal BMD (osteopenia/OP) group

**Figure 4:**
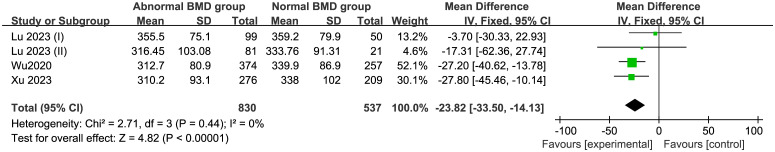
Forest of plot comparison of UA level in normal BMD group and abnormal BMD (osteopenia/OP) group.

Three studies comprising 1,367 patients were included in this meta-analysis. Compared with the control group, the abnormal BMD group had a MD of −23.82 μmol/L (95% CI −33.50 to −14.13; I^2^ = 0%; P=0.44) in a fixed model, indicating a lower UA level in the abnormal BMD group than that in the normal BMD group (**Figure 4**) Egger’s test indicated no publication bias (P = 0.175) (**Supplementary Figure S3**).

#### 3.4.4 Comparison of UA level in the osteopenia and OP groups

**Figure 5:**
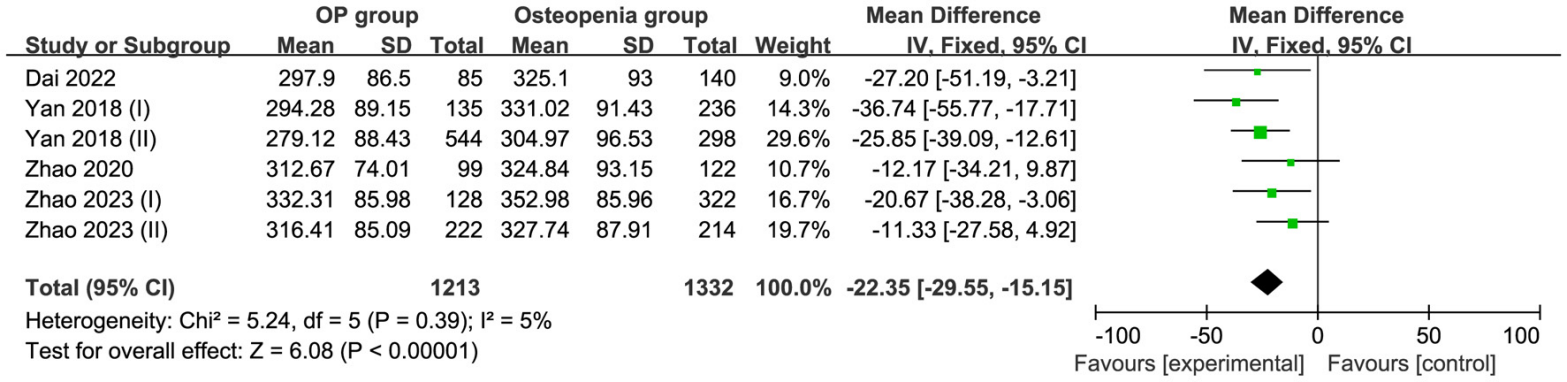
Forest of plot comparison of UA level in the osteopenia and OP groups.

Five studies comprising 2,545 patients were included in this meta-analysis. Compared with the osteopenia group, the OP group had a MD of −22.35 μmol/L (95% CI −29.55 to −15.15; I^2^ = 5%; P=0.39) in a fixed model, suggesting that the UA levels were higher in the osteopenia group than in the OP group (**Figure 5**). Egger’s test indicated no publication bias (P = 0.858) (**Supplementary Figure S4**).

#### 3.4.5 Comparison of UA level in the non-osteoporosis and OP groups

**Figure 6:**
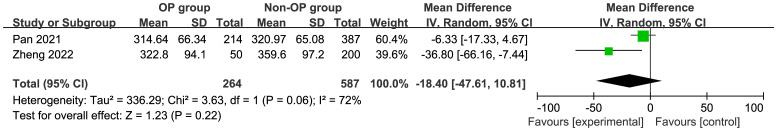
Forest of plot comparison of UA level in the non-osteoporosis and OP groups.

Two studies comprising 851 patients were included in this meta-analysis. Using a random effect model, the total effect yielded an MD of −18.40 μmol/L (95% CI −47.61 to 10.81; I^2^ = 72%; P=0.06) (**Figure 6**).

## 4 Discussion

Many pathogenic factors are associated with OP, including vitamin D deficiency, calcium deficiency, nutritional imbalance, aging, and oxidative stress. The impact of oxidative stress on bone metabolism may be mediated through the following pathways: upregulation of the receptor activator of NF-kB ligand and downregulation of osteoprotegerin, thereby increasing the generation of osteoclasts; reduction of osteoprogenitor cell differentiation into the osteoblast lineage, accompanied by an increase in pro-oxidants; reduced osteoblast activity; and increased osteoblast and osteocyte apoptosis (16).

Natural antioxidants, including UA, bilirubin, and albumin, are present in humans, among which UA is the most abundant. It can eliminate two-thirds of free radicals in plasma, and its antioxidant effect is greater than that of other enzymatic antioxidants (17). The mechanisms of UA that influence may be involved in several aspects. First, as the progenitor cells for bones, bone marrow-derived mesenchymal stem cells (BMSCs) have the capability to differentiate into osteoblasts. UA promotes the osteogenic differentiation of BMSCs through the increasing of the expression of Runt-related Transcription Factor 2 (RUNX2)/Core-Binding Factor Subunit Alpha-1 (CBF-alpha-1) gene (18). Second, Wnt signaling pathway can also affect bone remodeling. The expression of Wnt-3a and β-catenin was enhanced with increasing UA concentration, which may lead to increased osteoblast differentiation (19, 20). Third, UA downregulated the expression of 11β-Hydroxysteroid-Dehydrogenase-type-1 (cortisone reductase) enzyme. Thus, UA could promote hormone induced BMSCs differentiation into osteoblast (18, 21). Since OP is related to oxidative stress, the enhancement of antioxidant effects may prevent or stop the occurrence of OP or delay the progression of the disease.

Several studies have investigated the relationship between UA levels, oxidative stress, and OP. UA levels in postmenopausal women are lower than that in their perimenopausal counterparts, and this difference affects the development of OP (22). In a study by the National Health and Nutrition Survey cohort in the United States from 2005 to 2010, no significant correlation was found between UA levels and BMD in women ≥ 30 years of age. In animal experiments in the same study, similar results were obtained when comparing BMD between hyperuricemic and normal UA rats, with no significant differences reported (23). Among the 328 postmenopausal women, there was no significant relationship between the UA levels and BMD of the spine and femoral neck (17).

The target population of the present study was patients with T2DM because OP is currently considered to be one of several complications of diabetes (24). In addition to the macrovascular and microvascular complications of diabetes, bone metabolism is also an important complication that leads to disability and mortality. Our study found that among female patients, there was no significant difference in UA levels between the osteopenia or OP groups and the normal BMD group. UA levels were significantly lower in the abnormal BMD group than in the control group. Compared with osteopenia, UA levels in the OP group decreased significantly, indicating that we need to devote more attention to UA levels in clinical practice because some of the hazards of high UA levels, such as gout, have been established. However, if UA levels are too low, it may be related to OP, which suggests that an appropriate level of UA is necessary.

Some of the results of our study showed high heterogeneity, which may be due to the following factors: First, in terms of meta-analysis, the number of our studies and subjects involved in this meta-analysis were small which may lead to final results; Second, although subjects involved were T2DM, there were some differences at baseline, such as blood glucose level and lipid level, which may also affect heterogeneity.

The present study had some limitations, the first of which was its observational design, which precluded the determination of a causal relationship between UA, BMD, and OP. As such, interventional studies are necessary to establish such relationships. Second, some studies divided UA into 3 or 4 equal parts. Owing to the different grouping data, it was exceedingly difficult to compare the incidence rates of OP and osteopenia at different UA levels. Third, all studies included in this meta-analysis were conducted in China; a such the results cannot be generalized to all races and geographical regions. Fourth, based on existing results, we are currently unable to determine which UA level is most suitable for patients with OP or osteopenia; therefore, more rigorous studies with larger sample sizes are warranted. Last, advanced age is a risk factor for OP. After menopause, women experience a significant acceleration in bone loss due to a rapid decline in estrogen levels. Although men do not have a distinct turning like menopause, their testosterone levels gradually decrease with age, which leads to gradual bone loss. However, since the meta-analysis included studies that only provided the mean age and did not explicitly subgroup by menopausal status, we were unable to a stratified analysis.

## 5 Conclusion

Among female patients with T2DM, compared with the normal BMD group, there was no significant change in UA levels in the osteopenia and OP groups. Compared with the control group, patients with abnormal BMD (including osteopenia and OP) exhibited significant decrease UA levels in patients with T2DM. Furthermore, compared with the osteopenia group, the OP group exhibited a significant decrease in UA levels in T2DM. In clinical practice, for T2DM, we not only focus on blood glucose, blood lipids, and blood pressure, but also on UA level. On the one hand, UA level cannot be too high so as to avoid serious conditions such as gouty arthritis. On the other hand, it cannot be too low to avoid osteopenia and OP. However, what level of UA is appropriate needs further study.

## Data Availability

The original contributions presented in the study are included in the article/**Supplementary Material**. Further inquiries can be directed to the corresponding author.

## Supplementary material

Supplementary Figure 1 Egger’s publication bias plot of UA level in the normal BMD group and OP group.

Supplementary Figure 2 Egger’s publication bias plot of UA level in normal BMD group and osteopenia group.

Supplementary Figure 3 Egger’s publication bias plot of UA level in normal BMD group and abnormal BMD (osteopenia/OP) group.

Supplementary Figure 4 Egger’s publication bias plot of UA level in the osteopenia and OP groups.
