# Supplementary figures and images for: Correlation between uric acid levels and bone mineral density in patients with type 2 diabetes mellitus: a systematic review and meta-analysis
Source: Front Endocrinol (Lausanne). 2025 Feb 7;16:1415550. doi: 10.3389/fendo.2025.1415550 (PMC11842257; doi:10.3389/fendo.2025.1415550)

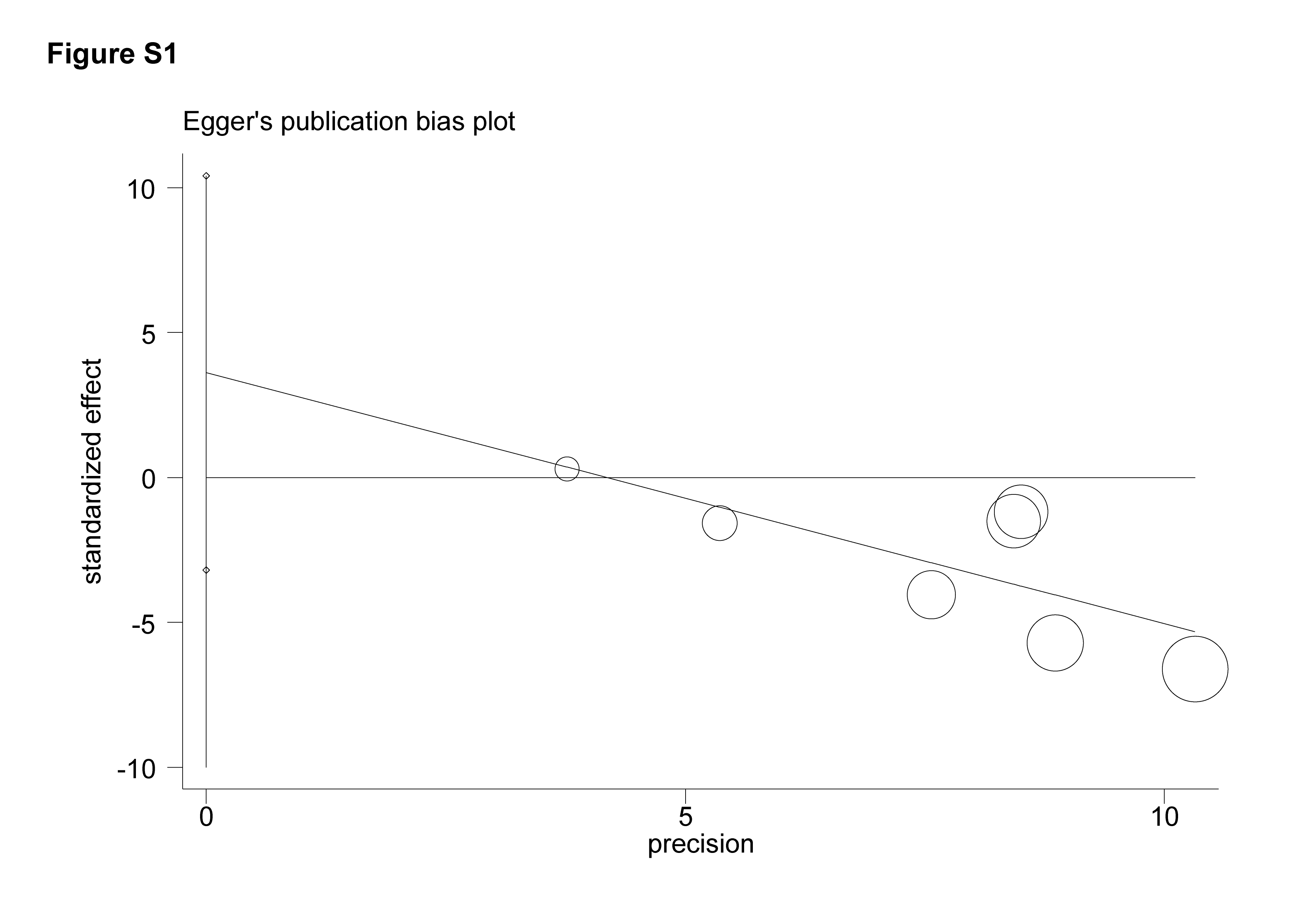

Supplement: Supplementary Figure 1 — Egger’s publication bias plot of UA level in the normal BMD group and OP group. [file Image1.tif]

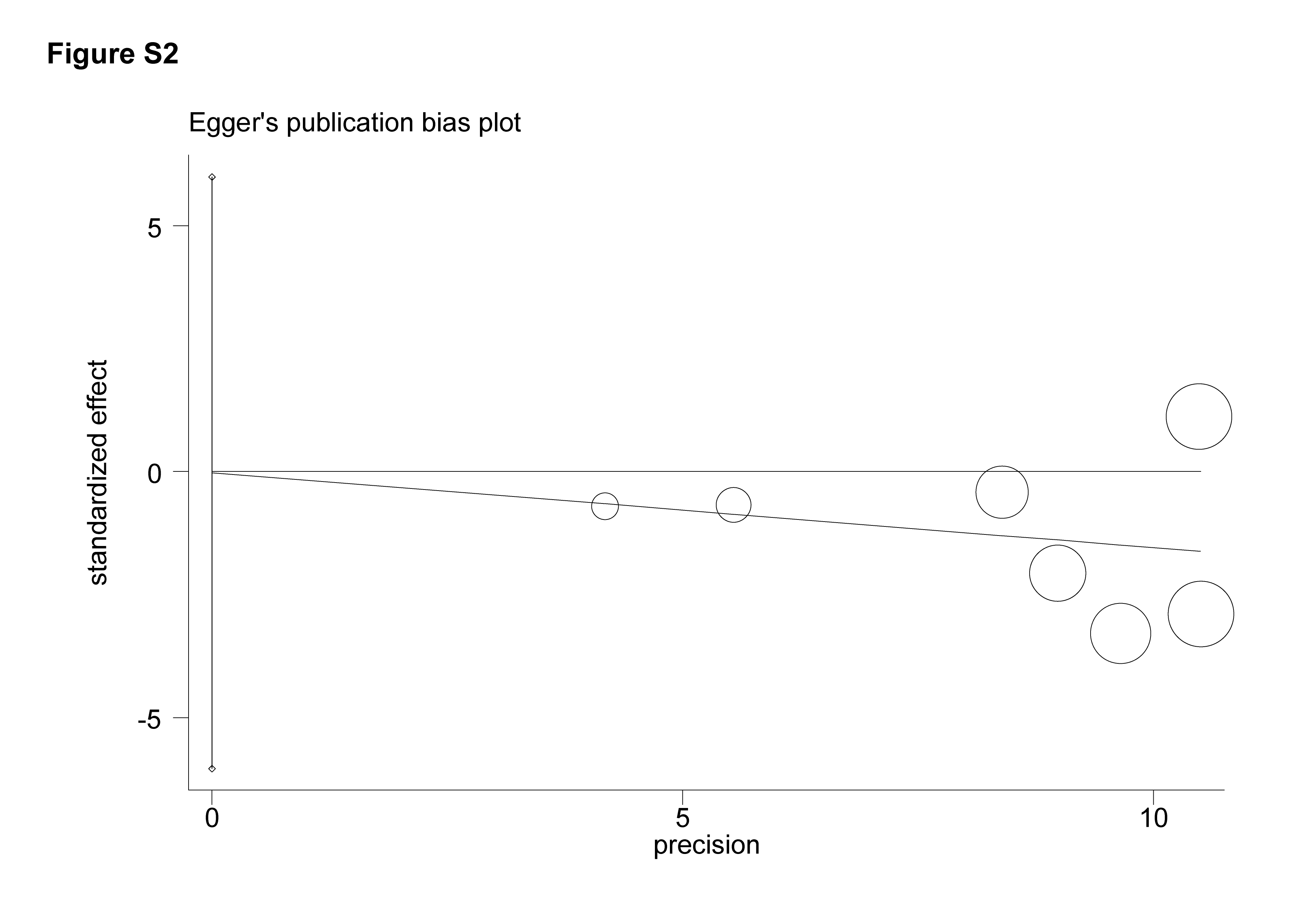

Supplement: Supplementary Figure 2 — Egger’s publication bias plot of UA level in normal BMD group and osteopenia group. [file Image2.tif]

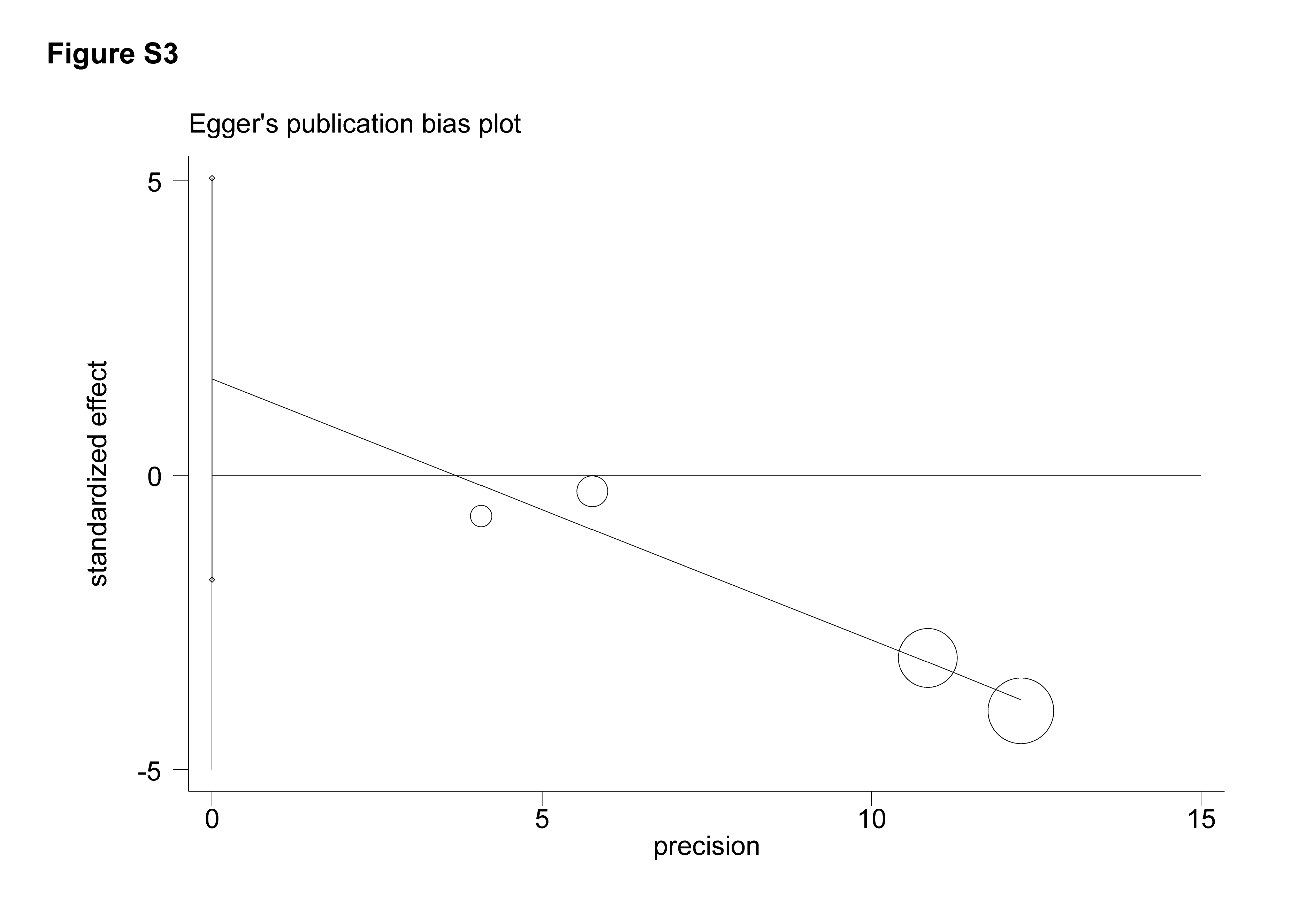

Supplement: Supplementary Figure 3 — Egger’s publication bias plot of UA level in normal BMD group and abnormal BMD (osteopenia/OP) group. [file Image3.tif]

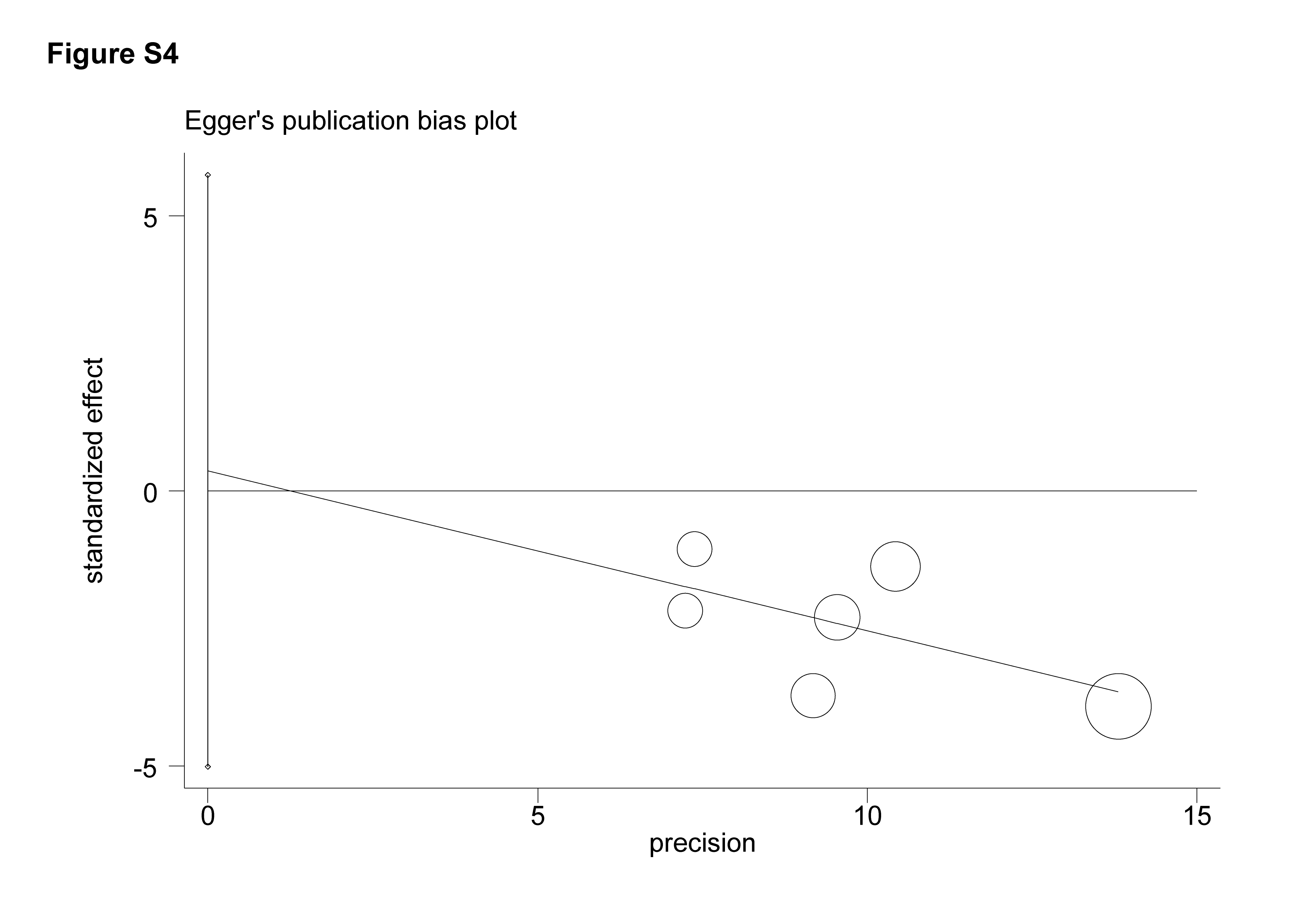

Supplement: Supplementary Figure 4 — Egger’s publication bias plot of UA level in the osteopenia and OP groups. [file Image4.tif]
